# Minimally-Invasive Assisted Robotic Spine Surgery (MARSS)

**DOI:** 10.3389/fsurg.2022.884247

**Published:** 2022-06-06

**Authors:** Ramiro A. Pérez de la Torre, Siddharth Ramanathan, Ashley L. Williams, Mick J. Perez-Cruet

**Affiliations:** ^1^ Department of Neurosurgery, Oakland University William Beaumont, School of Medicine, Royal Oak, MI, United States; ^2^ Michigan Head and Spine Institute, Southfield, MI, United States

**Keywords:** robotic, minimally invasive spine surgery (MISS), mazor X, stereotactic transformation, minimally-invasive robotic spine surgery (MARSS), da Vinci

## Abstract

Minimally-Invasive robotic spine surgery (MARSS) has expanded the surgeons armamentarium to treat a variety of spinal disorders. In the last decade, robotic developments in spine surgery have improved the safety, accuracy and efficacy of instrumentation placement. Additionally, robotic instruments have been applied to remove tumors in difficult locations while maintaining minimally invasive access. Gross movements by the surgeon are translated into fine, precise movements by the robot. This is exemplified in this chapter with the use of the da Vinci robot to remove apical thoracic tumors. In this chapter, we will review the development, technological advancements, and cases that have been conducted using MARSS to treat spine pathology in a minimally invasive fashion.

## Introduction

Spine surgery has experienced tremendous innovation and evolution over the last 50 years, including the implementation of novel technologies, the development of new procedures, and the expansion of biologics. Image guided surgery is one such technique that was developed due to a need to improve surgical precision and accuracy in complex cases. As image guided surgery has become more widely available, these technologies have been applied to the field of robotic surgery (1–5). Initially robotic surgery was a method to translate a virtually planned procedure into a localized surgical process, as seen in stereotactic cranial surgery. Many elements impact the fidelity of robotic surgery, including meticulous case selection, optimizing the method of pre-operative imaging, and 10–16, collaborating with industry to develop these systems. Starting in 2000, several adaptations in robotic and stereotactic systems were made that have led to the development of robotic interfaces that are currently being used to treat spine pathology (6).

One of the factors that prompted the development of robotics in spine surgery was the relatively steep learning curve of minimally invasive spine approaches. Due to the manual dexterity required to operate effectively within a narrow working corridor, manual minimally invasive spine surgery presents a unique challenge (7–9). However, there are certain procedures where the application of robotics presents a niche opportunity to improve surgical accuracy and efficiency, such as placement of percutaneous pedicle screws (10–16).

Consequently, image guided spine surgery has become a valuable tool for performing minimally invasive spine surgeries (17, 18). Several commercial systems have become available for cranial and spine procedures, with thousands of units being used in centers across the globe (18–20). The first reported robotic application in the neurosurgical field was for stereotactic brain biopsy utilizing the PUMA robot system. (PUMA 200) (6, 21) De Souza published the first spine robot in practice using the spine assist system (Mazor Robotics Ltd., Caesarea, Israel), which received FDA approval in 2004 (22). In 2008, the application of robotics in spine surgery was expanded with approval of NeuroMate (Integrated Surgical Systems, Sacramento, California, US). As the interest in these systems grew, further advancements utilizing tele-surgical robots including da Vinci (da Vinci Technologies) were developed (23).

**Figure 1:**
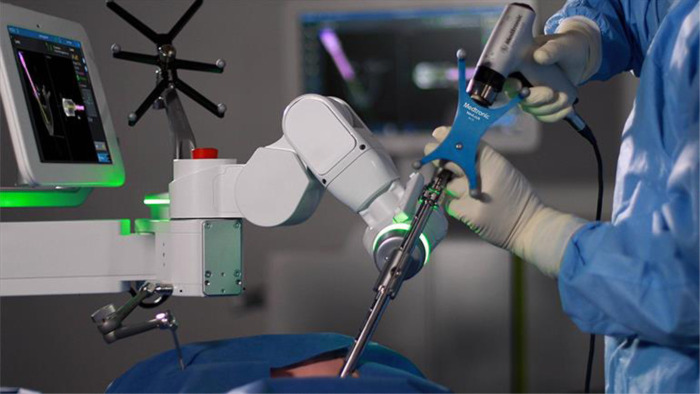
Photo of Mazor X (Mazor Stealth technologies, Medtronic). A commercial system designed to extend the working options, including imaging processing and robot-based interaction.

**Figure 2:**
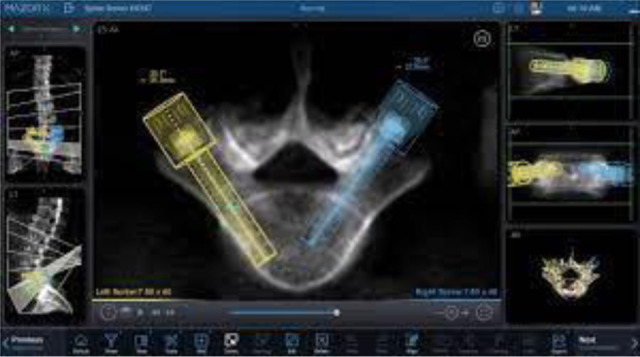
Software interaction with Mazor X, including pedicle screw selection along with optimization of construct definition. Proper pedicle screw diameter and length can be selected to conform to the patient's individual anatomy based on pre-operative imaging using the work-station.

**Figure 3:**
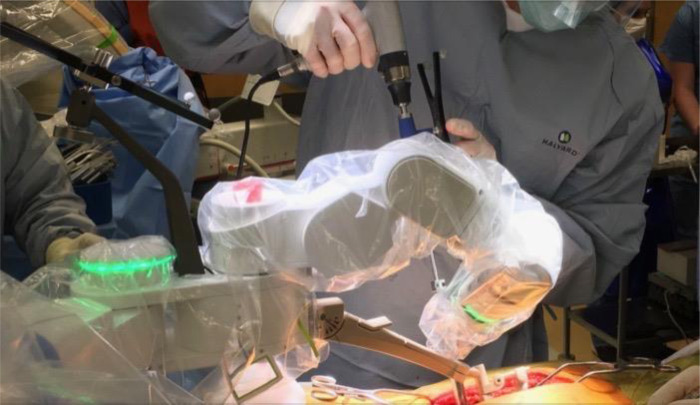
Mazor X in working position during drilling of the pedicle for pedicle screw application.

**Figure 4:**
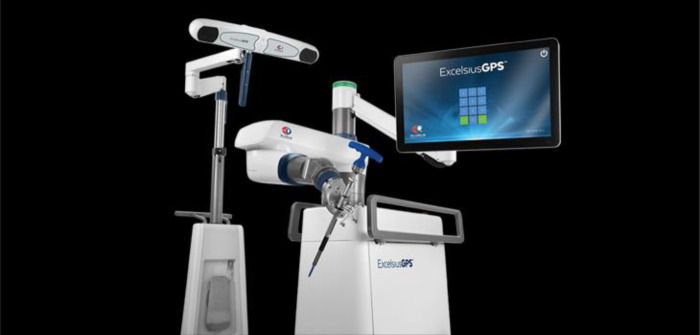
Excelcius GPS equipment. A robotic arm-based technology along with multiple intuitive functions for working environment.

**Figure 5:**
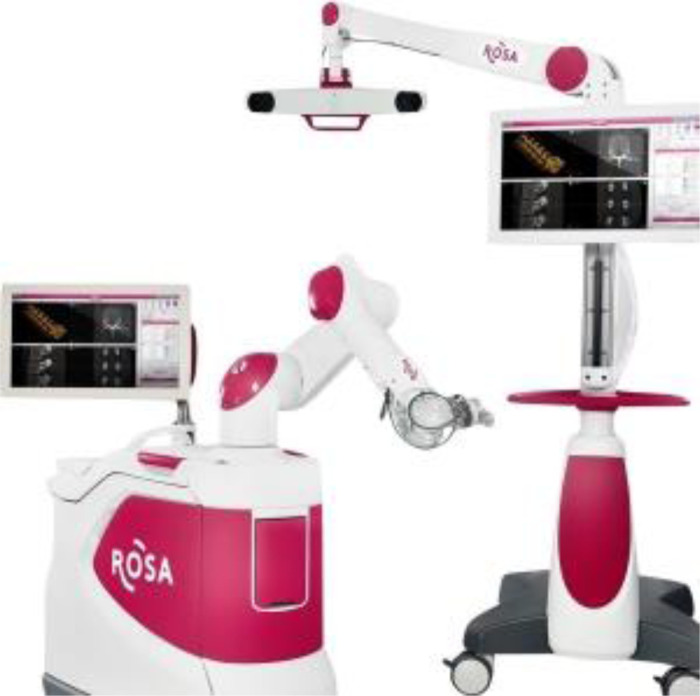
ROSA technologies (Zimmer Spine Inc.).

In the United States, there are several commercial spine robots available. These include the Mazor X, (Mazor X Stealth Station, Medtronic), Globus XPS (GPS Excelsius GPS® Robotic Navigation Platform | Globus Medical) and Rosa technologies (ROSA ONE® Brain-Zimmer Biomet) (Figures 1–5).

These technologies have progressed from simple robotic interfaces to minimally-assisted robotic spine surgery (MARSS). There is a growing interest in the use of robotics with telepresence systems, including the da Vinci robot. Joseph et al reported the use of robotics in spinal instrumentation and identified variables such as precision of screw placement, surgeon learning curve, radiation exposure, and reasons for robotic failure, making note of the high degree of accuracy that can be achieved using robotic instrumentation techniques (24). These aspects of robotic surgery underscore the growing relevance of robotic techniques in the treatment of spinal pathology.

## Methodology

Most of the robotic approaches in thoracolumbar spine are built as ordered steps to establish an intuitive work flow. The preoperative imaging include X-rays, computer tomography (CT), and magnetic resonance imaging (MRI). Specific protocols are used to preset software capabilities to optimize this process. Initial software interactions allow the surgeon to plan the procedure utilizing common surface rendering, hybrid imaging selection, image fusion and trajectory definition. Once the proposed plan has been defined, the incision is made using the robotic assisted approach. When tumor resection is required, planning steps can be created to allow safe removal of the identified structure through a minimally invasive robotic method. Vasculature can be clearly delineated to augment the safety of the surgical approach. In thoracotomy, robotic approaches can identify safe paths of entry into the chest cavity, and multiple thoracoscopic ports can be created according to the intrinsic patient pathology. The entry point for pedicle screw placement, trajectory definition for patients with challenging anatomy, and rod, pedicle screw, and interbody cage selection can all be done through the robotic software without utilizing physical trial implants. Each robotic system, given its proprietary design, affords surgeons the freedom of choice to choose their preferred system.

## Mazor X- Technologies

The commercially available Mazor X -Stealth Edition represents one of the most advanced technologies in the field. A truly hybrid system, this instrument includes a combination of image guided surgery and robotic arm capabilities. Most of the robotic systems in use today follow a similar setup process as outlined below.

### Imaging Acquisition and Preoperative Planning

Pre-operative image acquisition is performed using fluoroscopic x-rays and computed tomography (CT). The images are then transferred to the Mazor X workstation where surgical planning software allows a multiplicity of functions, including 3D reconstruction and surgical rehearsal. These functions allow for vertebral pedicle measurements, anatomical pedicle angulation, and pedicle screw implant selection. The list of pedicle screws can be optimized using a series of planning steps and parameters including trajectories, measurements, and construct alignment. On the day of the procedure, the working plan can be transferred to the robotic guidance system. Recent adaptations allow the use of intraoperative CT scanning as well (O-Arm, Medtronic technologies) (Figures 1–3).

### Patient Setup, Preoperative Preparation

The conventional methodology of spine surgery is followed during preoperative preparation: the patient is placed under general anesthesia, transferred to the operating table (Jackson table), and adequately padded. The appropriate draping system for the robot is used, and following the surgical preparation and draping, a guidance device is attached to the patient’s spine or iliac crest. We routinely use electromyographic (EMG) neuromonitoring to record pertinent nerve root potentials during the surgical procedure. The operation is then carried out in a linear, step-wise fashion.

These subsequent steps of MARSS include:

- Placement of the robotic arm in the proper position.
- Obtaining additional AP and oblique views for registration and stereotactic transformation (these images allow surface matching with the preoperative imaging set).
- Activation of the surgical robot interaction.
- Placement of the surgical drill in position to start the procedure.
- Replacing the starting drill with a serrated drill.
- Removal of the retractor and replacement with robotic extender to be used for pedicle screw placement.
- Positioning a Kirschner pin into the drilled hole.
- Manual placement pedicle screws onto the robot following the defined trajectory.
- Tapping the proposed trajectory, and continuing with pedicle screw placement.
- Correction, reduction, compression done in a specific order.
- Additional decompression, osteotomies or rod tightening done as required.
- Bone fusion including decorticating and drilling along with use of bone substitutes.

## Excelcius Robotic Applications

The robotic positioning system (Excelsius GPS, Globus Medical, Inc. Audubon, PA) are compatible with several imaging modalities, including a preoperative CT, an intraoperative CT or fluoroscopy. As in any camera based-tracking technology, it is important to establish a patient’s reference base for calibration purposes (25). The steps that need to be followed are described below (Figure 4).

### Preoperative CT

A computed tomography (CT) scan of all spinal levels using 1 mm cuts is critical to cover the proposed surgical levels. All images are subsequently transferred to the workstation for planning and creation of the virtual environment. The CT data set is usually transferred into the robotic positioning system and registration is subsequently completed for all vertebral levels.

### Intraoperative CT Methodology

The stack of images along with the coordinate system are transferred to the planning module. (O-arm, Medtronic SNT, Louisville, CO, USA) The installed software allows multiple trajectories to be planned for pedicle screw insertion. The entry point, trajectory, pedicle screw selection and optimization are done using the planning module.

### Surgical Technique

The initial portion of the procedure requires foot pedal activation for robotic arm movement. Once the entry point is defined, a pointing tube connector can be applied. A stab incision is done accordingly. Fascia and soft tissue dissection allow the entry point and initial trajectory to be executed. A final position to entry point is marked, and an initial working drill is inserted to the proposed trajectory. Electrophysiological monitoring is continuously done. A pedicle screw is inserted using a simple passing to the planned trajectory. Once all pedicle screws are completed, rods are passed through the connecting incision. Bolts secure the rod to the construct. Intraoperative images can be done at any point to verify the positioning of screws and rods. Decompression can be completed along with interbody placement.

## Rosa System Robotic Approaches

The imaging process and software interaction follow the proprietary design. Each technology confers additional advantages and interactions that facilitate the working process. Rosa technologies encompass a family of robotic equipment with several years in the market that display some unique features useful in spine surgery. These technologies allow for 6 degrees of movement in the robotic arm once the planning process has been completed, and an advanced integrated software allows multiple intuitive functions to be applied during the planning of working trajectories (Figure 5).

## Da Vinci Robot Technologies

Since its introduction into the surgical arena in 2000, the Da Vinci robot (Da Vinci Technologies) has undergone a series of developments to expand the range of utilization (26, 27). The telepresence modality utilized by the Da Vinci robot makes it one of the most versatile and utilitarian surgical instruments (28). An increasing number of publications exist that aim to broaden the surgical applications of this instrument (29–32).

### Pre-Operative Planning

**Figure 6:**
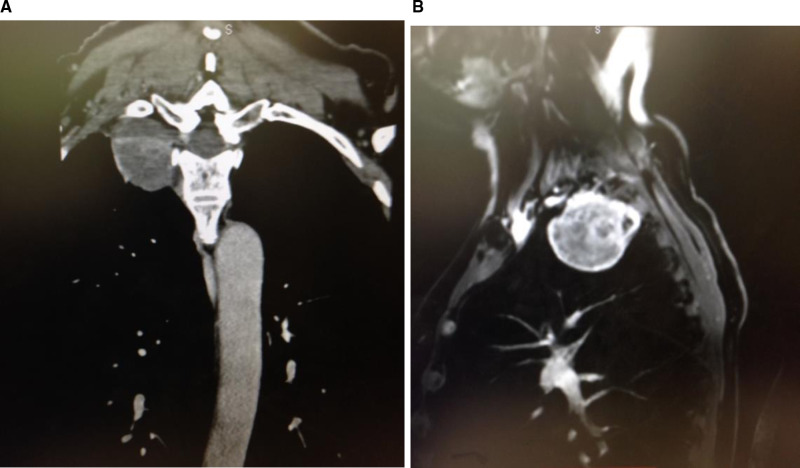
Pre-operative coronal and sagittal thoracic MRI images showing high apical chest tumor. Thin slice CT images can be very helpful in identifying the neural foramen of tumor origin. This is critical in safely detaching the tumor from the spinal canal before final removal through the chest cavity.

Patients typically present with apical thoracic lesions. The ideal patient in our opinion has well circumscribed lesions such at schwannomas or neurofibromas. As more efficient techniques for spine surgery using the da Vinci system are developed, the indications for utilizing this technology will expand accordingly. For patients presenting with apical thoracic schwannomas, imaging studies include contrast thoracic spinal MRI and CT to accurately identify the level of origin and determine the neural foramen from which the tumor originates (Figure 6).

A sagittal CT starting at the sacrum can help to accurately determine the level of the lesion. For determination of the proper surgical level, the following images are ordered: chest X-ray, thoracic and lumbar anteroposterior (AP) and lateral images. Intra-operative fluoroscopy is used to confirm the level of the lesion by counting vertebral bodies starting at the sacrum or ribs on the AP chest view. Alternatively, an opaque marker can be placed pre-operatively by interventional radiology to help identify the proper location of the tumor. In cases of removal of thoracic schwannomas, no implant instrumentation is needed.

### Surgical Technique and Case Examples

**Figure 7:**
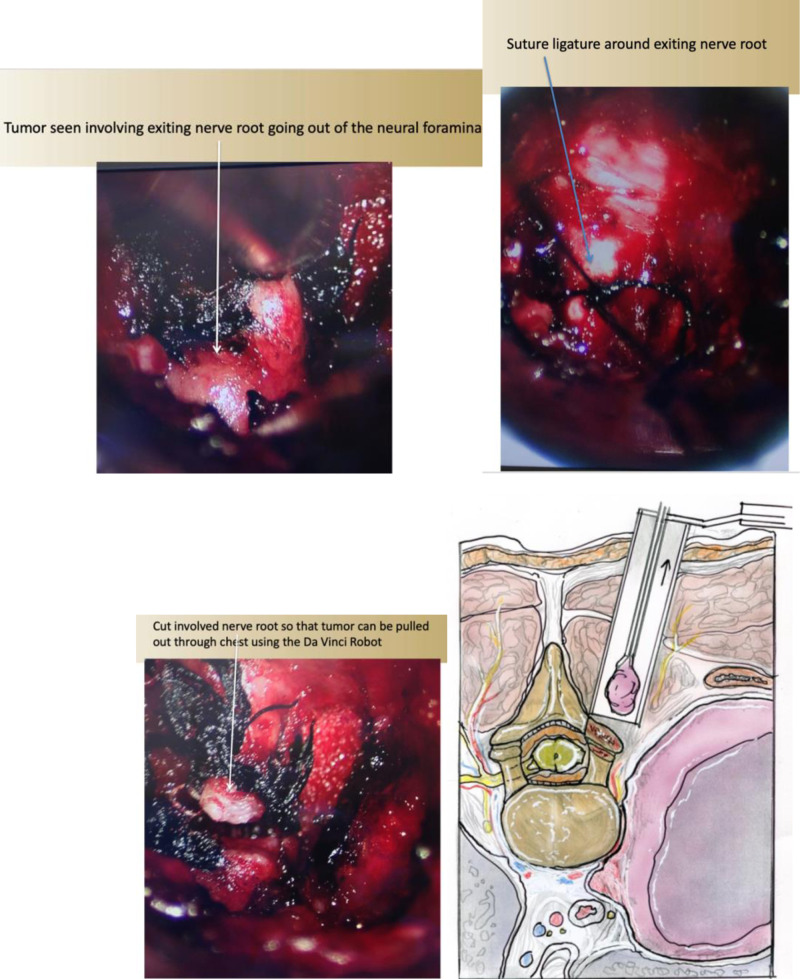
(**A**) Intra-operative photo showing (**A**) tumor extending outside the neural foramen, (**B**) silk suture ligature around the nerve giving rise to the tumor, (**C**) ligation of the nerve leading to the tumor. (**D**) Illustration of removal of intra-spinal canal portion of the tumor via a posterior approach through a tubular retractor.

The patient is initially positioned in the prone position on a Jackson table with all pressure points adequately padded. A Jackson table allows for unencumbered localization of the lesion using intra-operative fluoroscopy. Double lumen intubation is done to allow for collapse of the lung on the side used for the thoracic approach. Intra-operative electro-physiologic monitoring is used to measure somatosensory evoked potentials and motor evoked potentials. AP and lateral fluoroscopy are used to help localize the level. An incision is then made lateral to the midline based on pre-operative image analysis, and is typically only 2–3 cm from the midline. The fascia is cut, and a muscle dilating technique is used to approach the thoracic spine over which a tubular retractor is placed. Under microscopic visualization, the ipsilateral lamina and facet are exposed. A bone cutting drill with an M8 cutting burr is used to perform an adequate ipsilateral laminectomy and facetectomy, thereby exposing the tumor within the neural foramen and spinal canal. The contralateral aspect of the spine is not dissected. The drilled bone is collected using a BoneBac Press (Thompson MIS/Bonebac, Salem, NH) (10). This local morselized autograft bone is used to reconstruct the facet complex after tumor resection. The nerve root to the tumor, typically the sensory branch, is identified and ligated with silk ties (Figure 7).

**Figure 8:**
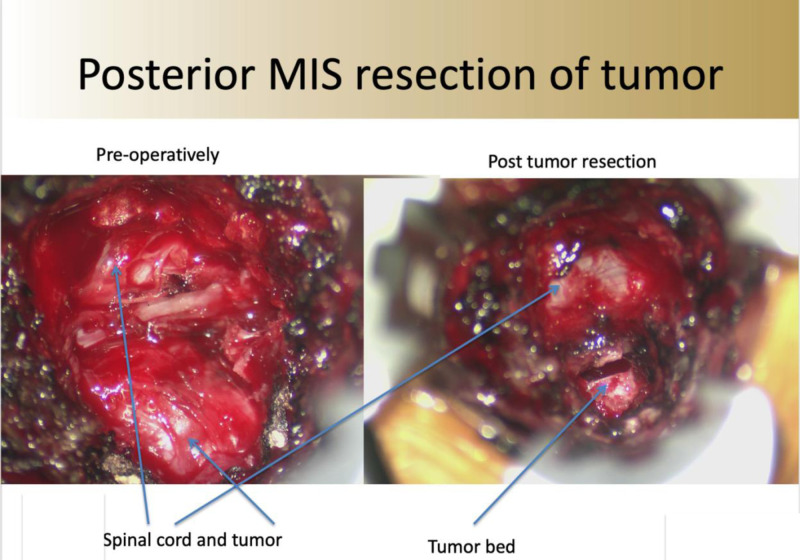
Intra-operative photos after minimally invasive posterior approach (**A**) tumor and intra-canal neural foramen tumor exposed and (**B**) after resection of the tumor with residual tumor bed.

The sheath of the tumor is opened and the tumor removed in a piecemeal fashion. To prevent potential cerebral spinal leakage into the thoracic cavity, the area can be covered with gel foam and thrombin sealant. Once complete hemostasis is achieved, the facet and laminectomy are reconstructed using the morselized autograft bone collected in the BoneBac Press. Gross total removal of the tumor extending into the spinal canal is achieved (Figure 8).

**Figure 9:**
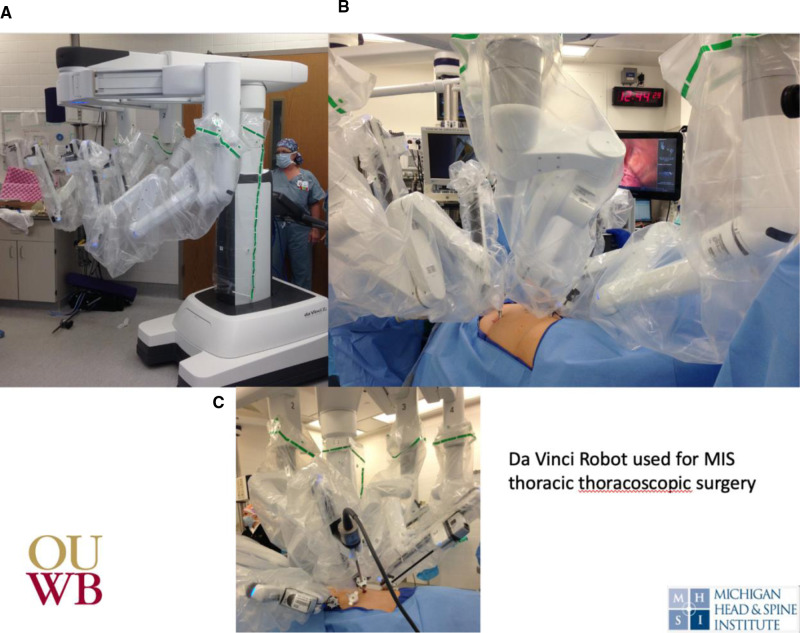
Intra-operative photos of (**A**) da Vinci robot, (**B**) used for anterior thoracoscopic approach the spine to (**C**) removal apical thoracic tumor.

The tubular retractor is removed, allowing the paraspinous muscles to return to their normal anatomic position. The fascia is closed using 2-0 interrupted Vicryl suture. A subcuticular interrupted suture is applied, and the skin incision is closed with skin glue.The patient is then repositioned in the lateral position on a sandbag to allow for adequate unilateral thoracic approach to the tumor. A thoracoscope can then be used for proper port placement. Thoracoscopic ports are placed and the De Vinci robot is positioned adequately (Figure 9).

**Figure 10:**
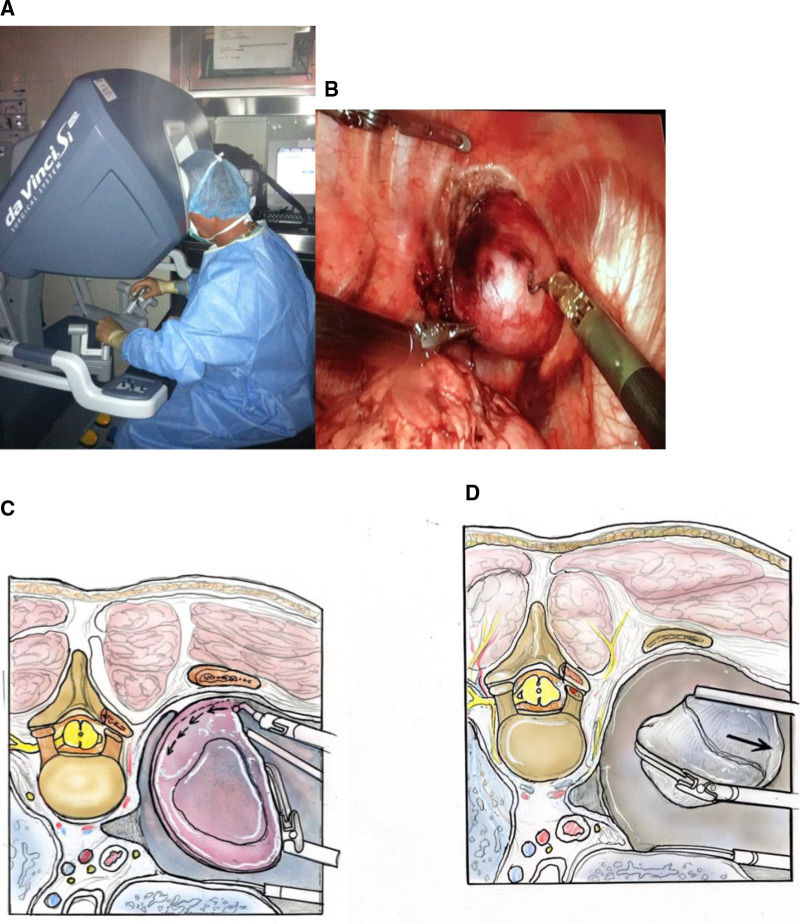
Intra-operative photo of (**A**) surgeon using the da Vinci robot to remove (**B**) apical thoracic tumor. Illustrations showing (**C**) resection of apical thoracic tumor from the chest wall and (**D**) removal of the tumor. (Illustrations from: An Anatomical Approach to Minimally Invasive Spine Surgery, Editors; M. Perez-Cruet, R. Fessler, M. Wang, Thieme Publishing Inc. NY, 2019).

Instruments are placed in the De Vinci robot for retraction of the tumor and cauterized removal of the tumor from the chest cavity. A separate port is used to place a suction to remove cautery smoke. Detaching the tumor from its spinal canal attachment allows for gross total removal and limits potential traction injury to the spinal cord. Once the tumor is resected, it can be placed into a gall bladder bag and removed via one of the thoracoscopic ports (Figure 10).

**Figure 11:**
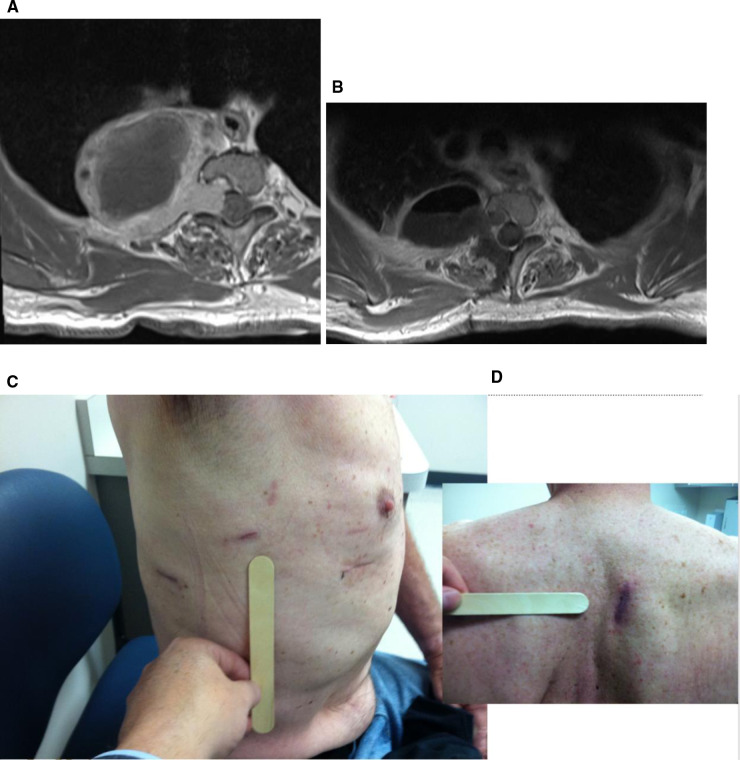
(**A**) Pre- and (**B**) post-operative axial MRI showing gross total tumor resection. Post-operative (**C**) anterior and (**D**) posterior thoracic incision after use of the da Vinci robot to remove apical thoracic tumor.

The thoracic ports are moved, a chest tube placed, and the incisions closed in the standard fashion. Re-inflation of the lung is performed before final closure. Patients are typically transferred to the intensive care unit for at least an overnight stay (Figure 11).

## Conclusion

While there are certainly challenges when using robots in spine surgery, there is a growing interest from the surgeon’s perspective to rely on robots due to the increased reproducibility, accuracy and precision. In the future, further technological advances could be integrated with robotic interaction to increase the ergonomic functionality of robotic instrumentation. Advances in artificial intelligence, big data use and haptics may all contribute to the continual improvement of robotic technology in spine surgery (33). Robotic systems such as Mazor XR, GPS Excelsius, and Rosa technologies have improved the ease and accuracy of surgical instrumentation placement. Tumors that are located in difficult positions can be resected using robotics in a minimally invasive fashion to improve outcomes and allow for more effective management of highly complex cases. MARSS or minimally- assisted robotic spine surgery represents a paradigm shift in spine surgery with the potential to revolutionize the field. As technologies evolve, we will continue to see broader applications of MARSS techniques in spine surgery with the capability of improving patient outcomes.
